# A Biosensor-CMOS Platform and Integrated Readout Circuit in 0.18-μm CMOS Technology for Cancer Biomarker Detection

**DOI:** 10.3390/s17091942

**Published:** 2017-08-23

**Authors:** Abdulaziz Alhoshany, Shilpa Sivashankar, Yousof Mashraei, Hesham Omran, Khaled N. Salama

**Affiliations:** 1 Computer, Electrical and Mathematical Science and Engineering Division (CEMSE), King Abdullah University of Science and Technology (KAUST), Thuwal 23955-6900, Saudi Arabia; yousof.mashraei@kaust.edu.sa (Y.M.); khaled.salama@kaust.edu.sa (K.N.S.); 2 Department of Biomedical Engineering, University of Chapel Hill/North Carolina State University, Raleigh, NC 27695, USA; ssivash@ncsu.edu; 3 The Integrated Circuits Lab, Faculty of Engineering, Ain Shams University, Cairo 11535, Egypt; hesham.omran@eng.asu.edu.eg

**Keywords:** biosensor, interdigitated electrodes, SSAT enzyme diagnostic platform, capacitive sensor interface, early cancer detection, CMOS

## Abstract

This paper presents a biosensor-CMOS platform for measuring the capacitive coupling of biorecognition elements. The biosensor is designed, fabricated, and tested for the detection and quantification of a protein that reveals the presence of early-stage cancer. For the first time, the spermidine/spermine N1 acetyltransferase (SSAT) enzyme has been screened and quantified on the surface of a capacitive sensor. The sensor surface is treated to immobilize antibodies, and the baseline capacitance of the biosensor is reduced by connecting an array of capacitors in series for fixed exposure area to the analyte. A large sensing area with small baseline capacitance is implemented to achieve a high sensitivity to SSAT enzyme concentrations. The sensed capacitance value is digitized by using a 12-bit highly digital successive-approximation capacitance-to-digital converter that is implemented in a 0.18 μm CMOS technology. The readout circuit operates in the near-subthreshold regime and provides power and area efficient operation. The capacitance range is 16.137 pF with a 4.5 fF absolute resolution, which adequately covers the concentrations of 10 mg/L, 5 mg/L, 2.5 mg/L, and 1.25 mg/L of the SSAT enzyme. The concentrations were selected as a pilot study, and the platform was shown to demonstrate high sensitivity for SSAT enzymes on the surface of the capacitive sensor. The tested prototype demonstrated 42.5 μS of measurement time and a total power consumption of 2.1 μW.

## 1. Introduction

Limited access to health-care systems and less healthy lifestyles in third world countries emphasize the need for low-cost point-of-care diagnostic systems [1]. Many drugs available in the market are designed to kill, attack, or at least slow the growth of cancer cells. In short, they are poisons with accompanying side effects. To avoid these side effects, many other cancer therapies have been pursued. One of the latest therapies for cancer includes metabolomics–based tests. Metabolomics, one of the “omic” sciences in systems biology, is the global assessment and validation of endogenous small-molecule biochemicals (metabolites) within a biologic system [2]. The metabolomics-based test consists of a single, one-shot application of amantadine, a drug which is already approved in both the US and Canada, after which a urine sample is collected two to four hours later and then analyzed. Acetyl amantadine (AcAm) is recognized as an exogenous cancer biomarker because it is the product of a metabolic process that is known to be significantly upregulated in cancerous cells. After ingestion, the anti-Parkinson and anti-viral drug amantadine is acetylated in the body by the enzyme spermidine/spermine N1 acetyltransferase (SSAT) to give AcAm, which can be detected in patient urine. Enzymes have been quantified using solid phase extraction (SPE) and tandem liquid chromatography with mass spectrometry (LCMS) from urine samples [3]. Raman spectroscopy is employed in medical diagnostics due to its ability to characterize and identify molecules with an inherent specificity [4]. Further, sensitivity in quantification is improved by Surface Enhanced Raman Scattering (SERS) [5]. However, techniques previously used to quantify AcAm in urine, such as liquid chromatography-mass spectrometry (LC-MS), are undesirable for clinical adoption due to high costs and long run times [6]. Therefore, there is a need to develop low-cost, fast, and portable diagnostic platforms to screen for the SSAT enzyme.

The SSAT enzyme production varies from person to person depending on the diet and lifestyle of the person. If the concentration of SSAT enzyme in the blood is higher than the normal production of theses enzymes then further diagnostics are required to precisely detect cancer progress. Almost all cells produce polyamines that include spermidine and spermine, but the increased amounts are prevailed in rapidly multiplying cells [7]. To show a dynamic range and sensitivity, a study on a population of different regions needs to be carried out to provide statistical analysis. Our aim is to provide an early-stage cancer detection system rather than monitoring the progression of cancer. As a pilot study, various concentrations were screened from 10 to 1.25 mg/L of the SSAT enzyme.

Recently, diagnostic platforms have been widely used to measure various types of analytes. These platforms include DNA analysis [8,9,10], protein quantification [11,12,13,14,15], and bacteria/cell growth monitoring [16,17,18]. Beyond these, CMOS biosensors are promising, as they can provide outstanding performance in sensing element and signal processing.

In in vitro diagnosis, different transducing mechanisms have been introduced for protein quantification. Optical sensing is reported in [13]. The photodetectors are used to transduce the optical properties from the fluorescence labels into an electrical signal. However, an excitation light source, filtering, and sophisticated sample pre-processing are required which increase the complexity of the biosensor and the power consumption. Another technique is to use an electrode or impedance sensor for enzyme/protein detection [19,20]. However, a potentiostat or network/impedance analyzer is utilized for interfacing the biosensor which is high-cost, large-sized, and complicated. Another approach that uses magnetism sensing has been proposed [12,21]. Hall sensors have been employed to transduce the magnetic field to voltage/current signals, and giant magnetoresistive (GMR) sensors are used in resistive sensing of the magnetic field. However, this type of sensing needs magnetic-particle labeling and magnetically sensitive transducers for which the GMR sensors are not available in standard CMOS technology. Moreover, signal amplification and conditioning are needed for the sensed signals, which increase the power consumption of the biosensor. Capacitive biosensors are promising as point-of-care devices due to their simple, low-cost structure, rapid detection, and portability. Moreover, capacitive sensors do not consume static power, and can be co-integrated with CMOS circuitry. Therefore, they are very attractive for use in battery-powered systems. Capacitive biosensors for protein detection are proposed in [22,23], where an LCR meter has been used for capacitance measurements. The LCR meter is a bulky, complicated, and expensive instrument. However, energy efficient integrated capacitance-to-digital converter circuitry can digitize the capacitance measurements with very low power consumption [24,25,26,27,28,29].

In this paper, we introduce an in vitro diagnostic biosensor-CMOS platform to screen the SSAT enzyme for low-power, rapid detection, and low cost. The sensing element is implemented based on capacitive sensing. It has a large sensing area with small baseline capacitance (femtofarad range) by connecting the capacitors in series, which increases the sensitivity and reduces the power consumption on the side of the readout circuit. The readout circuit is realized by a capacitance-domain successive-approximation (SAR) technique. It uses an inverter-based circuit that allows supply-voltage scaling for low-power requirement. Moreover, the readout circuit uses a clocked inverter-based quantizer for cascaded amplification by turning cascaded quantizers off when they are not in use. Thus, the average power of the quantizer is reduced. A binary-weighted capacitive digital-to-analog converter (DAC) is implemented by using a coarse-fine architecture to cover the wide dynamic range of the SSAT enzyme concentrations while occupying a small chip area.

The remainder of this paper is organized as follows. Section 2 provides a detailed description of the materials and the capacitive sensing element. Section 3 presents the readout circuit architecture and operation. Measurement results are discussed in Section 4, and the conclusion is drawn in Section 5.

## 2. Materials and Capacitive Sensing Element

### 2.1. Capacitive Biosensor

**Figure 1:**
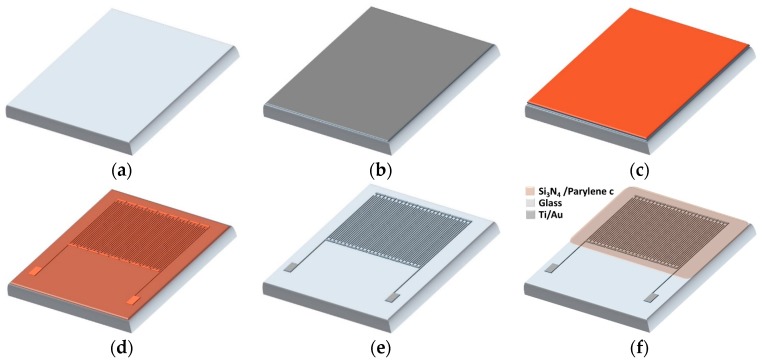
Fabrication of capacitive sensing element: (**a**) Clean glass substrate; (**b**) Sputtering of 10 nm Ti and 400 nm Au; (**c**) Photoresist coating; (**d**) Patterning of photoresist; (**e**) Photoresist develop, dry etching, and photoresist removal (**f**) Dielectric deposition S_i3_N_4_ (50 nm)/Parylene C (300 nm).

The fabrication process of the capacitive sensing element is shown in Figure 1. A glass substrate of 500 μm thickness is submersed in a hot piranha solution, then washed with distilled water and dried in a nitrogen drier. Ten nanometer (10 nm) titanium (Ti) and 400 nm gold (Au) metal layers are deposited by sputtering at 5 mTorr vacuum with 400 Wdc energy. The photolithography process is used to pattern the layers. Next, a dry etch process utilizing argon (Ar) gas at 300 W plasma power is used to etch the exposed metal to form the electrodes, then the photoresist is removed by using acetone and Isopropyl Alcohol (IPA).

**Figure 2:**
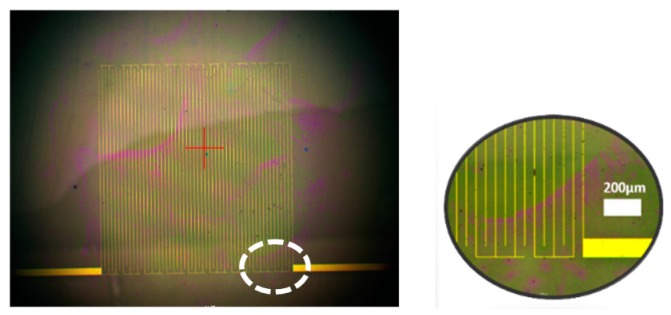
The sensor micrograph.

The capacitive sensor is a 9-segment array, as shown in Figure 2f. Each segment was realized with interdigitated electrodes fabricated on a glass substrate. The thickness of the metal finger is 410 nm with 50 μm of inner spacing between the fingers. The dielectric layer is then deposited by chemical vapor deposition (CVD) to form a 50 nm silicon nitride layer. After that, the capacitive sensor is coated with a 300 nm thin film of Parylene C to enhance the isolation and to keep it in the dynamic range of the readout circuit after depositing the analyte. The measured baseline capacitance of the sensor, which has a 2.92 mm × 3 mm area to cover the droplet of the biology sample, is 26 fF. The micrograph of the sensor is shown in Figure 2.

### 2.2. Sample Sites

The following materials were used for surface modification of the sensor and experiments. SSAT antibody was purchased from Santa Cruz Biotechnology, Inc. (Dallas, TX, USA) [30]. Spermidine-spermine+N1-Acetyltransferase+1+SAT1 was purchased from Insight Biotechnology. Tween-20, glutaraldehyde, and bovine serum albumin (BSA) were purchased from Sigma.

### 2.3. Surface Modification and Antibody immobilization

**Figure 3:**
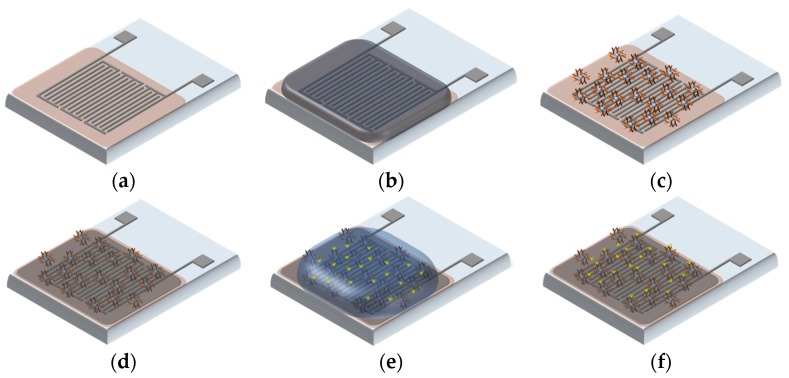
Procedure of antibodies immobilization: (**a**) Coated with 10 μL glutaraldehyde and incubated with Argon Gas for 2 h; (**b**) Coated with 2 μL antibody for 1 h; (**c**) Immobilized antibodies; (**d**) 2 μL of 2% BSA was added and incubated for 30 min; (**e**) Added antigen (10, 5, 2.5, 1.25 mg/L); (**f**) Antibody and antigen interaction.

The detailed procedure of the antibodies immobilization on Parylene C is shown in Figure 3. The cleaned surfaces of the capacitive sensors were incubated in 5% glutaraldehyde solution (cross-linking agent) in 1× PBS (phosphate buffer saline) solution (pH 7.4) and kept for 2 h in an inert argon atmosphere inside an enclosed chamber. Argon gas aids in absorbing active agents on the treated surface and covalently couples the active agent to the surface of the substrate [31]. The most common group present on the exterior of a protein is lysine with amino groups. Glutaraldehyde helps in the crosslinking of proteins to proteins or proteins to a surface-immobilized substrate due to the presence of its two amine functional groups [32].

After 2 h of incubation, the surfaces were thoroughly washed with PBS Tween-20/deionized (DI) water. A drop-cast method was used to apply antibody (2 μL) on the surfaces with a fine micropipette followed by incubation for 1 h at room temperature. The surfaces of the sensors were washed again in a similar manner and immersed in a 2% BSA solution as a blocking reaction for 30 min at room temperature to avoid any non-specific binding. After blocking, surfaces were again washed with the PBS Tween-20 buffer, which removes most of the unbound antibodies from the surfaces. Subsequently, the samples were incubated in the dark with a 2 μL solution of the SSAT enzyme. Both antibody and antigen conjugation were detected by the change in capacitance. The difference in capacitance corresponds to the concentration of the SSAT added after immobilization of the antibody. The capacitive biosensor can be reused with extensive cleaning and recalibration of the sensors. However, they get attached to the readout circuit via a simple connector and can be made disposable due to the reduced cost of manufacturing them [22,23,24,25,26,27,28,29,30,31,32,33,34], while the electronic readout system can be reused over and over. Moreover, the biosensors are designed to give quick readouts of the sample. Therefore, once the antibodies are immobilized on the surfaces, the stability is not of concern, as the antibodies do not degrade within a few hours. However, if the sensors have to be used for onsite detection, then they have to be shipped using Peltier coolers. Another approach is to eliminate certain factors that decrease the stability of biosensors [35]. Factors that affect the long-term stability of biosensors include (1) fouling and contamination of the samples; (2) the lifetime of the immobilized enzymes; and (3) strength of immobilization.

## 3. Successive Approximation Readout Circuit

There are several techniques for implementing the readout circuit for capacitive sensors such as incremental sigma-delta, period modulation, successive approximation, and dual slope [25,26,27,28,29]. The successive approximation readout circuit is adopted to interface the fabricated biosensor, as it achieves better energy efficiency.

**Figure 4:**
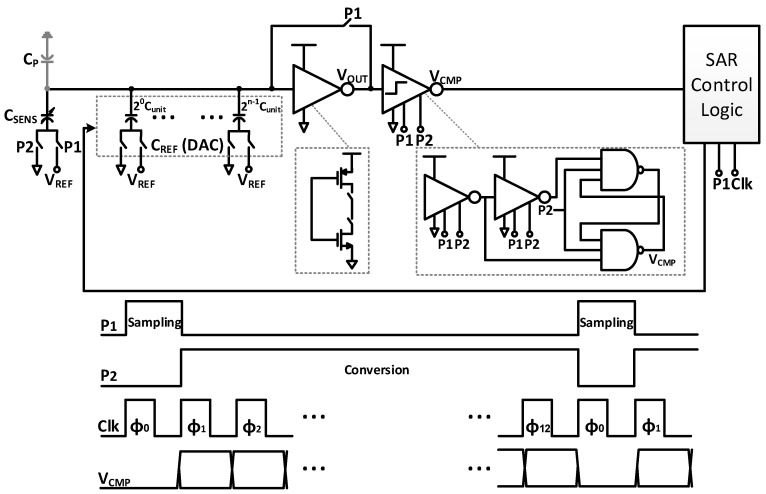
Readout circuit with associated timing diagram.

The schematic of the readout circuit is shown in Figure 4. It is based on a successive-approximation data converter that uses charge redistribution to produce a digital word. It consists of the unknown capacitive sensor (*C_SENS_*), a binary-weighted DAC (*C_REF_*), an amplifier with feedback loop, a single-ended comparator, and control SAR logic. The single-ended comparator consists of two cascaded inverters with clocked latch based on NAND gates. The amplifier and comparator (cascaded inverters) are implemented by using a clocked inverter. The circuit operation is divided into two phases: sampling phase and conversion phase. Two standard non-overlapping clock phases, P1 and P2, are required along with a SAR clock.

**Figure 5:**
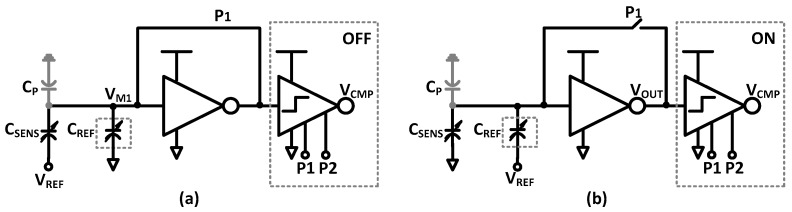
(**a**) Schematic and operation of sampling phase; (**b**) Schematic and operation of conversion phase.

To illustrate the readout circuit operation, Figure 5a shows the sampling phase of the readout circuit. When clock phase P1 is high, the amplifier is configured as a unity-gain amplifier by turning on the feedback switch. Thus, it employs the switching threshold voltage (*V_M_*_1_) to charge the binary-weighted DAC, parasitic capacitance ($C_{P}$), and the capacitive sensor (*C_SENS_*). The switches of the binary-weighted DAC are implemented by inverters. They are used to drive the bottom plates of the binary-weighted DAC to ground (GND) while the bottom plate of the sensing capacitor is connected to reference voltage (*V_REF_*). The single-ended comparator for cascaded amplification is turned off during this phase to reduce the average power consumption.

The second step is the conversion phase of the readout circuit, as shown in Figure 5b. In this phase, the clock P1 is low and the feedback switch is turned off. Thus, an open-loop amplifier is formed. Moreover, the bottom plates of the binary-weighted DAC are either connected to *V_REF_* or GND, depending on the N-bit SAR logic output. The equivalent capacitance of the elements connected to *V_REF_* and GND are *C_REF,ON_* and *C_REF,OFF_*, respectively, while the bottom plate of the sensing capacitor is connected to GND. The single-ended comparator for cascaded amplification is turned on during this phase. When the law of charge conservation is applied, the output voltage of the amplifier can be expressed as

(1) $$V_{OUT}=V_{M1}+\Delta \mathrm{Vo}=V_{M1}+\frac{(C_{SENS}-\;C_{REF,ON})\;V_{REF\;}}{C_{T}}A$$

where A is the finite gain of the inverter, *ΔV_O_* is the change in the output voltage, and $C_{T}$ is (*C_SENS_* + *C_REF_* + *C_P_*). Thus, the output voltage from the comparator is given by

(2) $$V_{CMP}=\{\begin{array}{c} \;0,\;\Delta Vo<0\\ 1,\;\Delta Vo>0 \end{array}$$

The successive-approximation algorithm keeps changing $C_{REF,ON}$ until it matches $C_{SENS}$ with an error less than 1 least-significant bit (LSB). The output voltage of the comparator is determined by the sign of (*C_SENS_* − *C_REF,ON_*), regardless of its absolute value (*ΔV_O_*). Thus, the circuit is insensitive to *V_REF_* and *C_P_* variation. When the mismatch between the amplifier and the comparator is considered in the analysis, the input voltage to the comparator is given by

(3) $$V_{IN}=V_{OUT}-\;V_{M2}=\frac{(C_{SENS}-\;C_{REF,ON})\;V_{REF\;}}{C_{T}}A+V_{OS}$$

where $V_{M2}$ is the switching threshold voltage of the comparator, and $V_{OS}$ is the offset voltage (*V_M1_* − V_M2_). Thus, the minimum achievable resolution is given by

(4) $$LSB=\frac{V_{OS}}{A\;V_{REF\;}}C_{T}$$

The LSB, gain, and full-scale range are therefore selected to limit the conversion error. For the implemented readout circuit, the gain is 98, and the offset voltage is 3.3 mV.

The binary-weighted capacitive DAC is used to compare the digital output with the capacitive input. The least-significant bit (LSB) capacitor is selected based on matching properties of the capacitors. Fringing capacitance between metal wires on the same layer is used to implement the fine binary-weighted DAC. Therefore, an 8-bit fine DAC with absolute resolution 4.5 fF is implemented. The total fine DAC capacitance (composed of 255 units) is 1.147 pF. The metal-insulator-metal (MIM) capacitor is used to implement the unit capacitor of the coarse binary-weighted DAC. It is chosen such that the DNL of the coarse DAC is less than 1/2 LSB of the fine DAC and the full scale of the fine DAC covers the unit capacitor of the coarse DAC. Therefore, a 4-bit coarse DAC with unit capacitor 1 pF is implemented and combined with fine DAC, which results in a 16.137 pF capacitance range in a compact area.

## 4. Measurement Results

**Figure 6:**
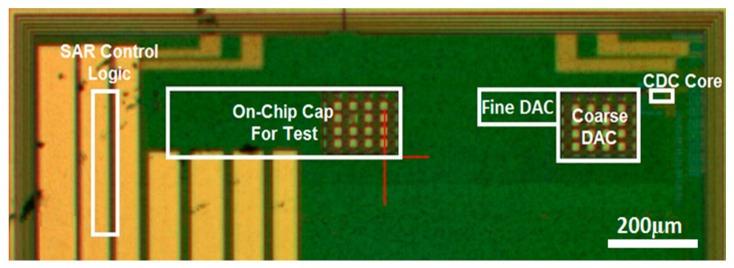
Chip photo of the prototype readout circuit in 0.18 μm CMOS process.

**Figure 7:**
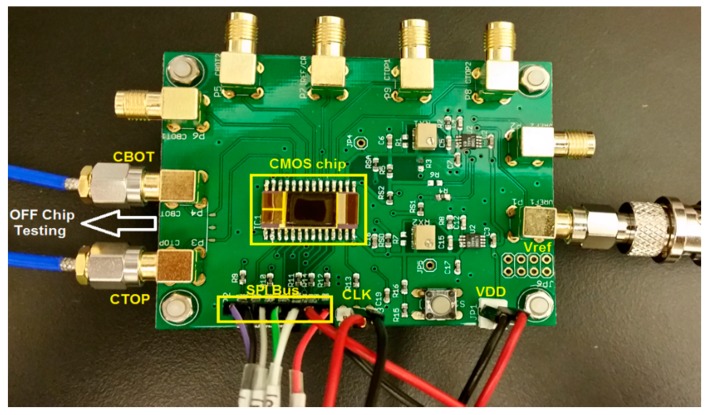
Test board for testing the packaged chip and the biosensor.

The prototype readout circuit was implemented in a standard 0.18 μm CMOS process. Figure 6 shows the chip photograph of the fabricated prototype. The active area is 0.06 mm^2^ including the binary-weighted DAC, analog circuitry, and SAR control logic blocks. A register file is used to read and write all digital signals through a 4-wire serial peripheral-interface (SPI) bus. The interface bus minimizes the bond pads and package pins. A printed circuit board was designed and fabricated to test the chip and the sensor. The chip was mounted on the circuit board and connected to the capacitive biosensor, as shown in Figure 7.

**Figure 8:**
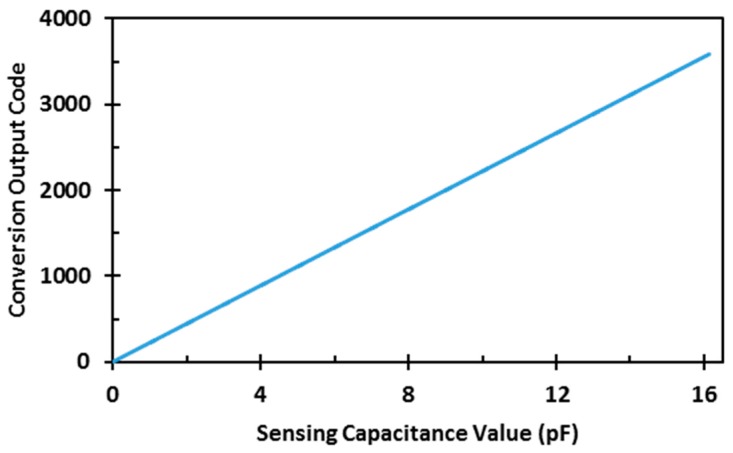
Measured transfer function of readout circuit.

**Figure 9:**
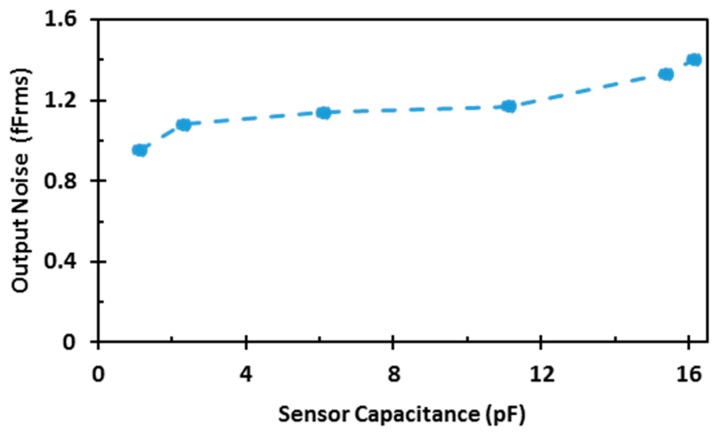
Measured noise output as a function of capacitance.

An on-chip dummy capacitive sensor is used to test the performance and the dynamic range of the readout circuit. The measured transfer function of the readout circuit is shown in Figure 8. The curve shows a linear relation between the output codes and input capacitances when the dummy capacitive sensor varies between 0 and 16.137 pF in 4.5 fF steps. The circuit is quantization-noise limited, and the noise is less than 1 LSB: i.e., it is limited by the smallest unit capacitor, as shown in Figure 9. It shows the standard deviation of the measured readout-circuit output as a function of sensor capacitance.

**Figure 10:**
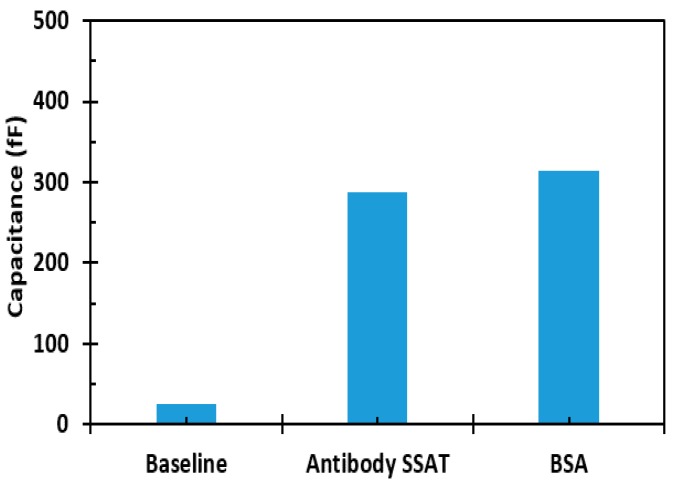
Measured baseline, antibody, and BSA capacitances of the sensor.

**Figure 11:**
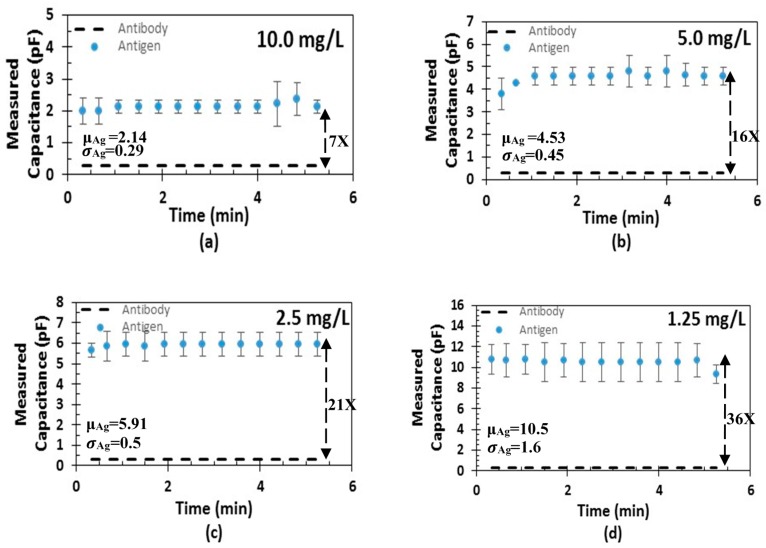
Sensor response to different concentrations of antigens: (**a**) 10 mg/L; (**b**) 5 mg/L; (**c**) 2.5 mg/L; (**d**) 1.25 mg/L.

**Figure 12:**
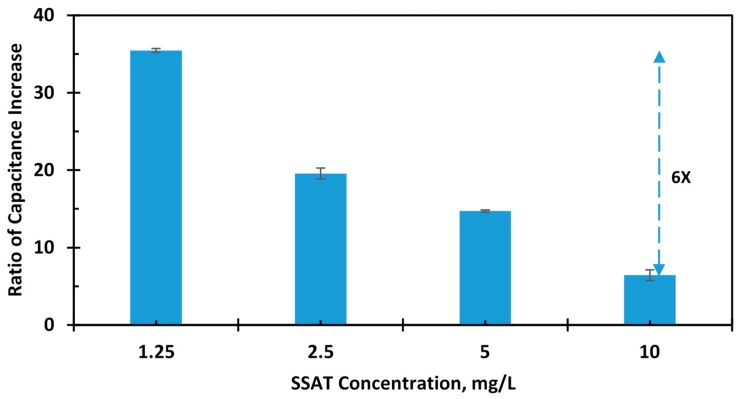
The ratio of increase in capacitance with various concentration of the SSAT.

Acetylation is performed by the enzyme SSAT, which has been proven to be present in elevated levels in many cancers including lung [36], breast [37], prostate, melanoma, and gastrointestinal tract cancers [38]. The enzyme is always present in all mammalian cells; the difference is that cancer patients have a higher proportion of the enzyme than healthy subjects. Therefore, various concentrations of the antigen from 10 to 1.25 mg/L have been screened. The change in capacitance of the sensor is shown in Figure 10. The baseline capacitance of the sensors was about 26 fF while, the capacitance change was substantial (about 300 fF) upon adding antibodies and after immobilization. Further, upon adding BSA, the capacitance was unchanged or the change was negligible, showing the specificity of the immobilized antibodies. Figure 11 reports the sensor response to the different concentrations of the SSAT enzyme with respect to the baseline capacitance after antibody immobilization. The measurements show that the capacitance of the sensor increases with the decrease in concentration of the enzyme. The error bars show the variation in the change of capacitance for different samples. The mean and the standard deviation are shown for every concentration. The platform wakes up intermittently every 20 s to perform and digitize the measurement of the biosensor and then returns to sleep mode during the testing of the sample. The sensor shows the response and stabilization within 1 minute, which indicates a high sensitivity to SSAT enzyme concentrations. The average change in capacitance for the highest and the lowest concentration is 5 folds. Figure 12 shows the ratio of increase in the capacitance (C_Antigen_ − C_Antibody_/C_Antibody_) with concentration. The ratio of capacitance increase was around 35 times for the concentration 1.25 mg/L and decreased to 6 times for the concentration 10 mg/L. This clearly indicates that a wide range of enzyme concentrations could be quantified using the diagnostic platform. The platform can quantify and digitize the given sample as low as 1.25 mg/L to 10 mg/L in its dynamic range with sufficient margin for any offset or sensor capacitance variation.

Table 1 lists the performance of the readout circuit. It shows the measurement time and the average power for analog and digital circuits. At full-scale input capacitance, the total power consumption of the readout circuit is 2.1 μW, of which the analog represents 85.7% and the digital represents 14.3%.

Table 2 summarizes performance comparison with previously published Biosensor-CMOS platforms. The introduced Biosensor-CMOS platform shows the ability to screen and quantify the SSAT enzyme on the surface of the capacitive sensor. It is based on capacitive sensing, which is low-cost and label-free. Moreover, the platform achieves an ultra-low power consumption of 2.1 μW among the published platforms, which is four orders of magnitude less power than the state-of-the-art.

## 5. Conclusions

An in vitro diagnostic, biosensor-CMOS platform was developed to screen and quantify SSAT enzyme for rapid detection that is low power and inexpensive. It will benefit early detection of cancer, which dramatically improves the survival rate of cancer patients. A large sensing area with small baseline capacitance was introduced to implement a capacitive biosensor that shows a high sensitivity to SSAT enzyme concentrations. A successive readout circuit incorporating a clocked inverter-based SAR architecture was introduced to read and process the information from the biosensor. By using a clocked digital inverter as a quantizer, the readout circuit is able to achieve an ultra-low power consumption. The diagnostic platform can be miniaturized into a complete microsystem in a compact size by integrating the sensing electrode and the associated readout circuit in a single tiny package. Therefore, the miniaturized device can be produced in low-cost, small size, and mass-production technology.

## Figures and Tables

**Table 1:** Readout circuit performance summary.

Technology	CMOS 180 nm
Power Supply (Analog/Digital)	0.9 V/1 V
Absolute Resolution	4.5 fF
Capacitance Range	16.137 pF
Noise (rms)	<1.4 fF
Power (Analog)	1.8 μW
Power (Digital)	0.3 μW
Resolution	12 Bits
Measurement Time	42.5 μs

**Table 2:** Comparison to some of the pre-existing biosensor-CMOS platforms.

Reference	BIOCAS 2010 [11]	JSSC 2013 [12]	VLSI 2015 [13]	JSSC 2013 [21]	This Work
**Target**	Protein	Protein	Protein	Protein	Protein
**Technology (nm)**	350	180	65	180	180
**Sensing Parameter**	Impedance	Magnetism	Fluorescence	Magnetism	Capacitive
**Target Protein**	Protein G	Human serum albumin	Streptavidin	SLPI cancer marker	SSAT enzyme
**Transducer**	Au electrodes	Hall sensor	Photodetector	GMR sensor	Au electrodes
**Labeling**	Label-free	Magnetic particle	Qdot 800 fluorophore	Magnetic particle	Label-free
**Immobilization**	Yes	Yes	Yes	Yes	Yes
**Power**	84.8 mW	300 mW	66 mW	50.4 mW	2.1 μW
